# Immunophoretic rapid diagnostic tests as a source of immunoglobulins for estimating malaria sero-prevalence and transmission intensity

**DOI:** 10.1186/1475-2875-8-168

**Published:** 2009-07-22

**Authors:** Geoffrey S Williams, Clement Mweya, Laveta Stewart, George Mtove, Hugh Reyburn, Jackie Cook, Patrick H Corran, Eleanor M Riley, Chris J Drakeley

**Affiliations:** 1 Joint Malaria Programme, Kilimanjaro Christian Medical Centre, PO Box 2228, Moshi, Tanzania; 2 Department of Infectious & Tropical Diseases, London School of Hygiene and Tropical Medicine, Keppel Street, London WC1E 7HT, UK; 3 Joint Malaria Programme, Teule Hospital, PO Box 81, Muheza, Tanzania; 4 Biotherapeutics Group, National Institute for Biological Standards and Control, South Mimms, Herts EN6 3QG, UK; 5 National Institute for Medical Research, Tukuyu station. Tanzania

## Abstract

**Background:**

Sero-epidemiological methods are being developed as a tool for rapid assessment of malaria transmission intensity. Simple blood collection methods for use in field settings will make this more feasible. This paper describes validation of such a method, by analysing immunoglobulins from blood retained within immunophoretic rapid diagnostic tests (RDTs) for *Plasmodium falciparum*. RDTs are now widely used for the diagnosis of malaria and estimation of parasite rates, and this method represents a further use for these devices in malaria control.

**Methods:**

Immunoglobulins eluted from RDTs, designed to detect parasite histidine rich protein-2 (HRP-2), were analysed by indirect ELISA for IgG recognizing the *P. falciparum *blood stage antigens merozoite surface protein-1_19 _(MSP-1_19_) and apical membrane antigen-1 (AMA-1). Optimal storage conditions for RDTs were evaluated by comparing antibody responses from RDTs stored in dry or humid conditions at 4°C or at ambient temperature (with or without air-conditioning) for 7, 31 or 70 days. Antibody levels estimated using 3,700 RDT samples from attendees at health facilities in North-eastern Tanzania were compared with contemporaneously collected filter paper blood spots (FPBS) and used to estimate seroconversion rates.

**Results:**

Storage of RDTs at 4°C was optimal for immunoglobulin recovery but short-term storage at ambient temperatures did not substantially affect anti-malarial IgG levels. Results from RDTs were comparable with those from FPBSs, for both antigens. RDT-generated titres tended to be slightly higher than those generated from FPBSs, possibly due to greater recovery of immunoglobulins from RDTs compared to filter paper. Importantly, however, RDT-based seroconversion rates, and hence serological estimates of malaria transmission intensity, agreed closely with those from FPBSs.

**Conclusion:**

RDTs represent a practical option for collecting blood for sero-epidemiological surveys, with potential cost and logistical advantages over filter paper and other blood collection methods. RDT-based seroepidemiology can be incorporated into routine monitoring of malaria endemicity, providing information to supplement parasite prevalence rates and generating rapid, robust assessment of malaria transmission intensity at minimal extra cost.

## Background

Accurate assessment of malaria transmission intensity is important in order to focus, monitor and assess the effectiveness of malaria control initiatives. The 'gold standard' measure of malaria transmission intensity is the entomological inoculation rate (EIR), i.e. the number of infectious mosquito bites per person per year. Alternatively, sero-conversion rates can be calculated using age-specific anti-malarial antibody prevalence. This is based on observations that sero-conversion rates for malarial blood stage and sporozoite antigens correlate closely with levels of exposure to *Plasmodium falciparum *[1]. This sero-epidemiological approach has a number of advantages over established entomological methods, including speed, cost, and relative insensitivity towards short-term changes including seasonal variations in transmission [2]. The logistical challenge of collecting, transporting and storing blood samples in field studies can be reduced by using filter paper to collect samples from finger-pricks [3]. However, this is still a moderately time-consuming process, which has no direct benefit to participants. Therefore, this study investigated the potential to carry out malaria serological assays using blood samples retrieved from rapid diagnostic tests (RDTs), which are commonly used in field studies to diagnose malaria and assess the rates of patent parasitaemia within communities. This refinement would extend the operational utility of sero-epidemiological methods for rapid assessment of malaria.

**Figure 1:**
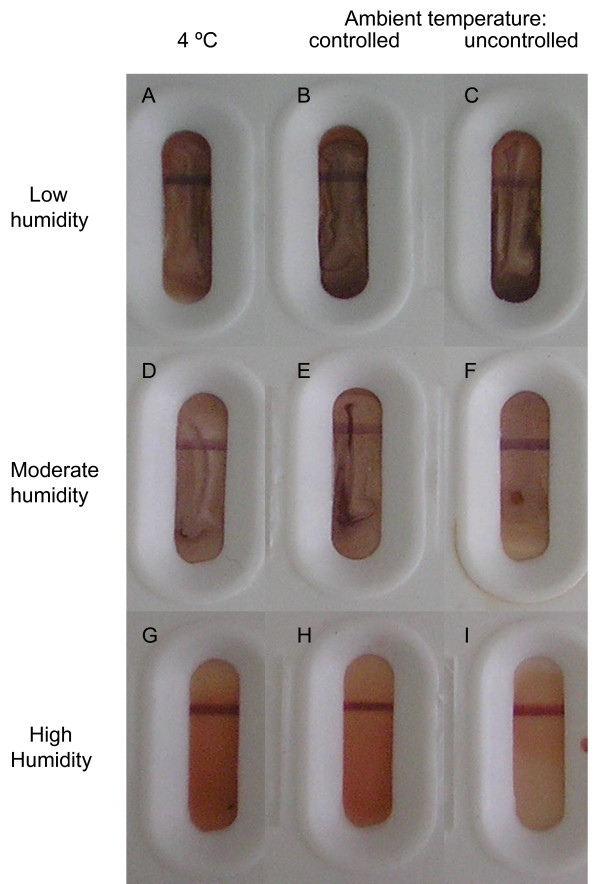
**Effect of storage conditions on appearance of *P. falciparum *rapid diagnostic tests (RDTs)**. Following their use in malaria diagnosis, immunophoretic RDTs for *P. falciparum *histidine rich protein-2 (HRP-2) were dried and stored with desiccant (A, B, C), dried and stored without desiccant (D, E, F) or stored with wetted tissue paper (G, H, I) at 4°C (A, D, G), or ambient temperature in a room which was either air-conditioned (B, E, H) or not (C, F, I) for 70 days and photographed. RDT test-strips were darker coloured after storage at lower humidity, while temperature had no appreciable effect on the appearance of RDTs. RDTs shown were for subject Afr4 and are representative of RDTs from all subjects.

RDTs are disposable diagnostic devices, which detect *P. falciparum *specific proteins (e.g. histidine-rich protein-2, HRP-2) in blood, and are becoming widely used for malaria diagnosis [4,5]. Their ease of use, relatively low cost and robustness make them attractive for use in resource-poor settings. Moreover, as the cost of malarial chemotherapy increases, RDTs are becoming more cost-effective, especially in areas of low transmission [6]. For tests based on HRP-2, the RDT consists of a plastic cassette enclosing absorbent pads and a nitrocellulose strip which contains immobilized and gold-labelled anti-HRP-2 antibodies [7]. The test is performed by the addition of blood (usually from a finger-prick) and a buffer solution to the devices; distinctive coloured lines appear on the nitrocellulose strip depending on whether *P. falciparum *HRP-2 is present in the blood or not (Figure 1).

Since blood is retained within RDTs, these devices represent a potentially useful source of immunoglobulins for sero-epidemiology. This would obviate the need to collect filter paper blood-spots or microtainer samples, simplify sample collection and storage, reducing costs and potentially increase acceptance. However, accurate serological data from RDTs can only be obtained if immunoglobulins are preserved in a functional state during storage and transportation. Temperature and humidity were shown to be key factors affecting the recovery of immunoglobulins from filter paper blood-spots [3], and so it is important to know how these parameters affect the recovery of immunoglobulins from RDTs. It is also important to check that serum proteins can be efficiently eluted from the devices, and that detection of human serum antibodies is not adversely affected by capture and detection antibodies with which the devices are impregnated during their manufacture and use. This study was conducted to evaluate the sensitivity, specificity and operational feasibility of using RDT-captured blood samples for sero-epidemiological estimation of malaria transmission intensity and have developed a protocol, which should prove suitable for use in the field.

## Methods

### Subjects and ethical approval

Ten ml heparinized venous blood samples were obtained from five patients (Afr1–Afr5) admitted to Teule Designated District Hospital, Muheza, Tanga Region, Tanzania which serves an area previously documented as highly endemic for *P. falciparum *[8,9]. Although febrile, none of the patients were showing signs of severe malaria, all had haemoglobin levels >11 g/dl, all were parasite negative by microscopy and were HIV sero-negative (Determine, Abbott Pharmaceuticals). Two healthy Europeans who reported they had never had malaria, and had never lived in a highly malarious area, were also recruited (Eur1, Eur2). These seven samples were used to determine the optimal storage conditions for RDTs.

To compare serological data from RDTs with that obtained from filter paper blood spots (FPBS), finger-prick blood samples were collected onto RDTs (Parahit-f, Span Diagnostics Limited, Sachin, Surat, India), alongside samples collected onto Whatman 3 MM filter paper (Whatman, Maidstone, UK) from all consenting attendees (patients – whether suffering from malaria or any other condition – and accompanying family members or guardians) at eight health facilities in the Korogwe and Same districts in north-east Tanzania. Korogwe district is a moderate malaria transmission area in Tanga region, while Same district is in Kilimanjaro region and is categorized as a low transmission site [10]. Samples were collected from approximately 4,000 individuals, 500 per health centre, over an eight week period in June and July 2007. Full details of the selection and recruitment procedures have been described elsewhere [11].

Ethical approval was obtained from the institutional review boards of the National Institute of Medical Research, Tanzania (NIMR/HQ/R.8a/Vol.IX/392 & NIMR/HQ/R.8a/Vol.IX./553), and the London School of Hygiene & Tropical Medicine (LSHTM: #2087 & 5136).

### Collection, storage and recovery of blood samples on RDTs

Prior to use, RDTs were stored at room temperature (not exceeding 35°C) according to manufacturer's instructions. All devices were in-date and opened immediately prior to use. For optimization of storage conditions for blood-exposed RDTs (i.e. after their initial use in malaria diagnosis), exactly 5 μl of heparinized blood was added to each RDT (using a micro-pipette), which was then incubated at ambient temperature and humidity for 20 min and the results interpreted according to manufacturer's instructions. RDTs were then either: air-dried for 2 h and stored with the desiccant from their original packaging (low humidity); air-dried and stored without desiccant (moderate humidity); or not air-dried and stored with wet tissue paper (high humidity). RDTs were then stored at 4°C or at ambient temperature with or without air-conditioning (ambient temperature – controlled or uncontrolled respectively), in sealed polypropylene boxes, for 7, 31 or 70 days.

For the health centre study, RDTs were performed according to manufacturer's instructions and the result of the test (i.e. parasite positive or parasite negative) was recorded. After air-drying, RDTs were kept at ambient temperature, in a sealed container with silica gel desiccant, for up to two weeks before being transfered to 4°C where they were kept until use. RDTs were dismantled and the blood-exposed components cut into small pieces, placed in 1.5 ml micro-tubes and immersed in 1 ml phosphate buffered saline (PBS) supplemented with 0.05% (v/v) Tween-20 (Sigma) (PBS/T) and 0.1% (w/v) Sodium Azide (Sigma). Tubes were incubated overnight (with agitation) after which RDT eluates were separated from solid RDT components, transferred to 96-well format deep-well plates (Greiner PS) and stored at -20°C until assayed. All eluates from the different storage conditions and different time points were assayed at the same time.

### Enzyme-linked immunosorbent assays (ELISAs)

Eluates from RDTs were analysed in duplicate for IgG recognizing the 19-kilodalton carboxy-terminal fragment of *P. falciparum *merozoite surface protein 1 (MSP-1_19_, Wellcome Genotype) [12] or apical membrane antigen 1 (AMA-1; 3D7 genotype) using indirect ELISAs as previously described [1,11]. Eluates from matched FPBS samples were analysed previously. Recombinant antigens were coated overnight at 4°C at a concentration of 500 ng/ml. Plates were washed with PBS plus 0.05% Tween 20 (PBS/T) and blocked with 1% (w/v) skimmed milk powder in PBS/T. RDT eluates were equivalent to a 1/200 dilution of whole blood or 1/400 of serum (assuming a haematocrit of approximately 50%). Samples were further diluted with PBS/T to give a final serum dilution on the processed ELISA plate equivalent of 1/1,000. Preliminary investigations indicated that best results were obtained when RDT eluates were incubated on ELISA plates for 3 h at ambient temperature. Pooled hyperimmune and malaria-unexposed serum (Human AB serum, Sigma) were run on each ELISA plate as positive and negative controls. After washing, horseradish peroxidase-conjugated rabbit anti-human IgG (DAKO) (1/5000 in PBS/T) was added to all wells. All plates were developed using OPD-hydrogen peroxide substrate solution, reactions stopped with 2 M H_2_SO_4 _and optical density (OD) values were read immediately at 492 nm.

### Statistical analysis

For each sample, duplicate OD values were averaged and normalized against the positive control for each plate. A threshold above which samples were deemed sero-positive was defined using a mixture model as previously described [3]. Briefly, the distribution of normalized OD values was fitted as the sum of two Gaussian distributions (a narrow distribution of sero-negatives and a broader distribution of sero-positives) using maximum likelihood methods. The mean OD of the Gaussian distribution corresponding to the sero-negative population plus three times the standard deviation was used as the cut-off for sero-positivity [3,13]. A separate cut-off was generated for each antigen (MSP-1_19 _and AMA-1) and cut-offs were generated separately for RDT-derived data and for FPBS-derived data. The sero-conversion rate (SCR or λ) was estimated by fitting a simple reversible catalytic model to the sero-prevalence – stratified into yearly age-groups – using maximum likelihood methods. For these models, only individuals aged one year or more were included, to remove the effect of maternally-derived antibodies. Additionally, evidence for temporal changes in SCR was explored by fitting models in which the SCR is allowed to change at a single time-point [11]. Statistical analyses were carried out using Stata 10 (Statacorp, Texas USA).

## Results

### Defining optimal RDT storage conditions

Following storage at different humidities and temperatures for 70 days, RDTs were visually inspected. RDTs stored within sealed boxes containing desiccant (Figure 1A–C), were darker coloured than RDTs stored without desiccant (Figure 1D–F), which in turn were darker coloured than those stored with wet tissue paper (Figure 1G–I). Storage temperature did not affect the appearance of RDTs noticeably (Figure 1A–I).

**Figure 2:**
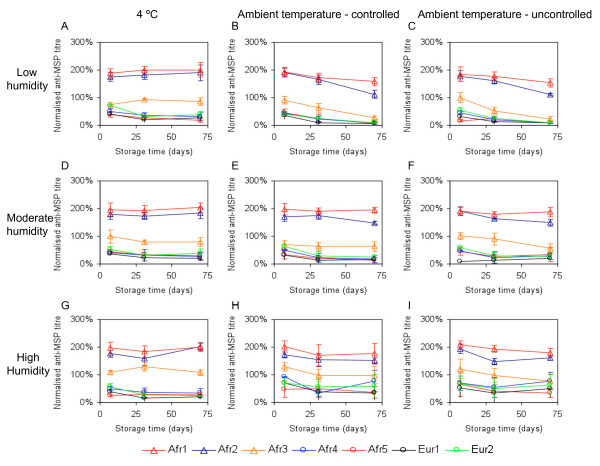
**Effect of RDT storage conditions on anti-merozoite surface protein (MSP)-1_1–19 _titres**. RDTs were stored at a range of humidities and temperatures for 7, 31 or 70 days and used a source of antibodies for estimation of anti-MSP-1_1–19 _titres using indirect ELISAs. RDTs were preserved optimally when stored at 4°C (A, D, G) and/or at moderate humidity (D, E, F). Values for high-titre samples decreased slightly with time for RDTs stored at ambient temperature and low humidity (B, C). Values for samples stored at ambient temperature and high humidity were less reproducible and for low-titre samples there were inappropriately high values detected for all time-points (H, I). Values are mean (+/- SD) optical density values from anti-MSP-1_1–19 _ELISAs, normalized against a standard sero-positive plasma pool, for three independent experiments.

Anti-MSP-1_19 _titres for two of the *Plasmodium*-exposed subjects (Afr1, Afr2) were high (Figure 2A–I). One (Afr3) had a moderate titre, while the two remaining subjects (Afr4, Afr5) had low titres, which were similar to those of the *Plasmodium*-unexposed Europeans (Eur1, Eur2). Irrespective of humidity, all samples stored at 4°C seemed to be well preserved, i.e. there were only small changes in the anti-MSP-1_19 _titre over time (Figure 2A, D, G). For samples stored at ambient temperature, those at moderate humidity also exhibited only small changes in titre over time (Figure 2E, F). For high-titre samples stored at ambient temperature and low humidity, there was a very small but reproducible reduction in anti-MSP-1_19 _titre (Figure 2B, C). Titres for samples stored at ambient temperature and high humidity were less reproducible between repeats of the assay, and for low-titre samples, inappropriately high anti-MSP-1_19 _titres were detected at all time-points (Figure 2H, I). Together, these data indicate that samples were best preserved when stored dry (with or without desiccant) at 4°C.

### Comparison of RDT and filter paper for collection and recovery of anti-malarial antibodies

**Figure 3:**
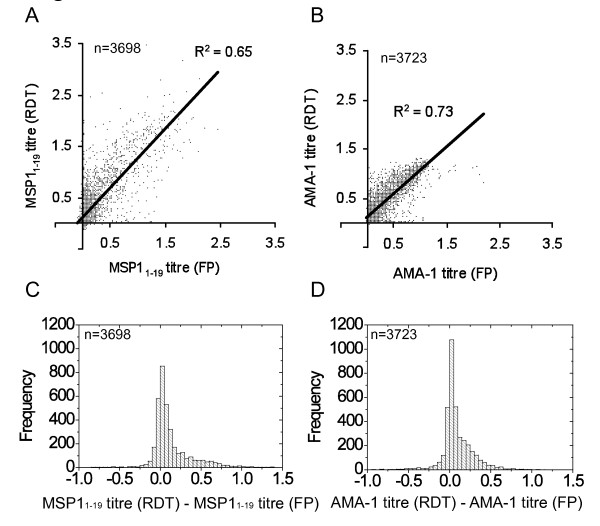
**Relationship between RDT and filter paper derived antibody titres to malaria antigens**. Anti-MSP-1_1–19 _and apical membrane antigen (AMA)-1 titres in attendees at eight health-care facilities in North-eastern Tanzania were determined using RDTs and compared to data from matched filter-paper blood spots [11]. Anti- MSP-1_1–19 _(A) and AMA-1 (B) titres determined using filter paper (FP) blood-spots and RDTs were closely correlated (*R*^2 ^= 0.65 for MSP-1_19_, *R*^2 ^= 0.73 for AMA-1). Panels C and D show the distribution of differences between RDT and filter paper titres for MSP-1_19 _and AMA-1 respectively.

Blood samples collected onto RDTs were tested for antibodies to MSP-1_19 _(n = 3,698) and AMA-1 (n = 3,723) and results were compared with data from matched samples collected onto filter paper. Antibody titres for samples recovered from RDTs and filter paper were highly correlated for both antigens (Figure 3A, B). Titres generated from RDT eluates were higher than those generated from filter paper for 75.1% of individuals in the case of MSP-1_19 _(Figure 3C) and for 77.2% of individuals for AMA-1 (Figure 3D). This difference likely reflects greater blood volumes, higher absorbance or more complete elution of immunoglobulins from RDTs compared to filter paper.

### Sensitivity and specificity of RDT versus filter paper for identification of sero-positive individuals

Individuals were classified as negative or positive for antibodies to MSP-1_19 _and AMA-1 using sero-positivity thresholds calculated separately for RDT- and FPBS-derived data. Since RDT-derived samples gave higher titres than FPBS samples, sero-positivity thresholds calculated for RDTs were higher than for FPBS for both MSP-1_19 _(0.245 vs 0.11) and AMA-1 (0.152 vs 0.069). The proportion of individuals defined as sero-positive by analysis of RDT-derived samples (32.3% for MSP1_19 _and 50.4% for AMA-1) or FPBS-derived samples (29.5% for MSP1_19 _and 50.8% for AMA-1) was similar.

**Figure 4:**
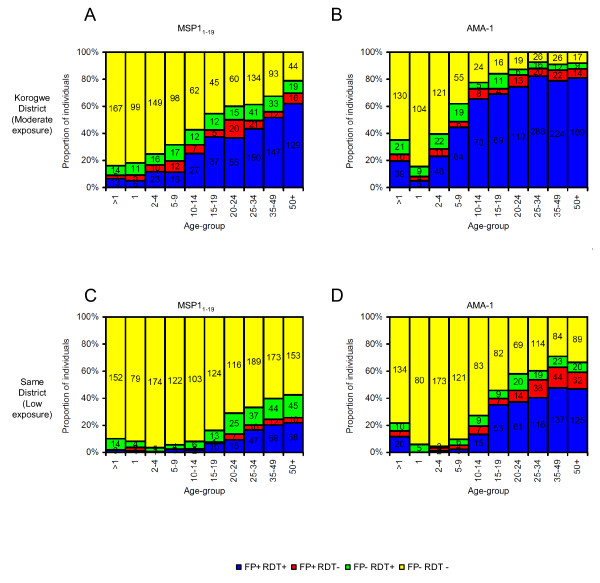
**Comparison of sero-positivity to malaria antigens for RDT- and filter paper-derived samples by age and transmission setting**. Individuals were defined as sero-positive or -negative for MSP-1_19 _(A, C) and AMA-1 (B, D) based on analysis of RDTs, and this data was compared to data from filter paper blood-spots [11]. Data were pooled from different sites within Same district (A, B; low malaria exposure) and Korogwe district (C, D; moderate malaria exposure). Incomplete data-points (i.e. only RDT or FP data available) were omitted. Most individuals were defined as seropositive or -negative by both FP and RDT-based serology (FP+ RDT+ and FP- RDT- respectively), while FP and RDT results were discordant (FP+ RDT- and FP- RDT+) for only a minority of individuals. Numbers of individuals are indicated on bars.

For the majority (85.2% for MSP-1_19 _and 85.9% for AMA-1) of individuals, filter paper and RDT results were concordant, i.e. these individuals were either defined as sero-positive by both approaches or defined as sero-negative by both approaches (Table 1 and Table 2). For both antigens, discordant samples were distributed broadly across age-groups and sites (Table 1, Table 2, Figure 4) and tended to result from samples giving values very close to the cut-off. For the purposes of calculating the sensitivity and specificity of RDT-derived sero-positivity data, FPBS data were used as the 'gold standard', i.e. were assumed to be correct. For MSP-1_19_, the sensitivity of RDT sampling was 79.0% (95% CI 76.5–81.4) and the specificity was 87.3% (CI 85.9–88.5), while for AMA-1 the sensitivity was 85.8% (CI 84.1–87.3) and the specificity was 86.0% (CI 84.3–87.6). Therefore, RDT-derived serological data agreed closely with FPBS-derived data.

**Table 1:** Comparison of seropositivity data obtained from either rapid diagnostic tests (RDTs) or filter paper (FP) blood-spots

Site	Age Group	MSP1_1–19_				
		
		Concordant		Discordant		
				
		FP- RDT-	FP+ RDT+	FP- RDT+	FP+ RDT-	Agreement
Overall		2336	801	387	156	85.2%
Korogwe	>1	167	13	14	5	90.5%
	1	99	6	11	5	86.8%
	2–4	149	23	16	10	86.9%
	5–9	98	16	17	12	79.7%
	10–14	62	27	12	7	82.4%
	15–19	45	37	12	5	82.8%
	20–24	60	55	15	20	76.7%
	25–34	134	150	41	21	82.1%
	35–49	93	147	33	12	84.2%
	50+	44	129	19	16	83.2%

Same	>1	152	3	14	0	91.7%
	1	79	1	4	2	93.0%
	2–4	174	1	5	0	97.2%
	5–9	122	3	4	0	96.9%
	10–14	103	2	6	1	93.8%
	15–19	124	10	13	1	90.5%
	20–24	116	15	25	7	80.4%
	25–34	189	47	37	10	83.4%
	35–49	173	58	44	12	80.5%
	50+	153	58	45	10	79.3%

Individuals were defined as sero-positive or sero-negative for MSP-1_19 _based on analysis of RDTs (this study) or contemporaneous FP blood-spots (as reported previously;[11]. Percentage agreement was calculated as the number of concordant individuals (FP-RDT- or FP+ RDT+) divided by the total number of individuals. Incomplete data-sets (i.e. only RDT or FP data available) were excluded from the analysis.

**Table 2:** Comparison of seropositivity data obtained from either rapid diagnostic tests (RDTs) or filter paper (FP) blood-spots

Site	Age Group	AMA-1				
		
		Concordant		Discordant		
				
		FP- RDT-	FP+ RDT+	FP- RDT+	FP+ RDT-	Agreement
overall		1567	1617	253	269	85.9%
Korogwe	>1	130	39	21	10	84.5%
	1	104	6	9	4	89.4%
	2–4	121	46	22	11	83.5%
	5–9	55	64	19	6	82.6%
	10–14	24	70	5	8	87.9%
	15–19	16	69	11	4	85.0%
	20–24	19	110	6	13	87.2%
	25–34	26	288	16	20	89.7%
	35–49	26	224	12	22	88.0%
	50+	17	169	9	14	89.0%

Same	>1	134	20	10	7	90.1%
	1	80	0	5	0	94.1%
	2–4	173	2	2	4	96.7%
	5–9	121	3	6	4	92.5%
	10–14	83	15	9	7	86.0%
	15–19	82	53	9	7	89.4%
	20–24	69	61	20	14	79.3%
	25–34	114	116	19	38	80.1%
	35–49	84	137	23	44	76.7%
	50+	89	125	20	32	80.5%

Individuals were defined as sero-positive or sero-negative for AMA-1 based on analysis of RDTs (this study) or contemporaneous FP blood-spots (as reported previously;[11]. Percentage agreement was calculated as the number of concordant individuals (FP-RDT- or FP+ RDT+) divided by the total number of individuals. Incomplete data-sets (i.e. only RDT or FP data available) were excluded from the analysis.

### Assessment of sero-conversion rates from RDT and FPBS samples

**Figure 5:**
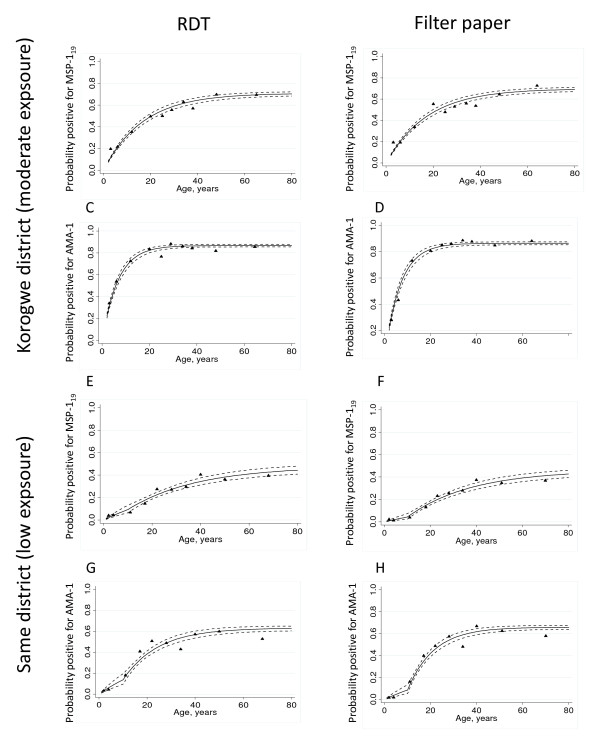
**Comparison of sero-conversion profiles between RDT- and filter paper-derived antibodies**. Age-specific sero-conversion profiles for MSP-1_19 _and AMA-1 were generated from RDT- and FPBS-derived blood samples. Panels A to D are for the Korogwe site and panels E to H are for Same. Plots A, B, E and F are for MSP-1_19 _and plots C, D, G and Ha are for AMA-1. Plots A, C, E and G are for RDT samples and plots B<, D, F and H are for filter paper samples. In each plot, black triangles represent observed data and solid black lines represent values from a catalytic conversion model. Dotted black lines represent the (95% CI).

Age-specific sero-prevalence plots for RDT-derived and FPBS-derived data, and the models fitted to these data, are shown for Korogwe and Same districts (Figure 5). On the basis of these fitted models, seroconversion rates were calculated (Table 3). There is strong agreement (*R*^2 ^= 0.96) between the sero-conversion rates calculated from RDT- and FPBS-derived samples (Table 3).

**Table 3:** Comparison of sero-conversion rates derived from RDT and from filter paper blood-spots

			RDT			Filter paper		
						
			Seroconversion rate (95% CI)			Seroconversion rate (95% CI)		
Korogwe	MSP-1_1–19_		0.042	(0.039	0.046)	0.040	(0.037	0.044)
	AMA-1		0.129	(0.117	0.143)	0.127	(0.116	0.140)
Same	MSP-1_1–19_	previous	0.018	(0.014	0.022)	0.019	(0.016	0.022)
		current	0.011	(0.007	0.016)	0.011	(0.007	0.016)
	AMA-1	previous	0.049	(0.040	0.060)	0.068	(0.058	0.080)
		current	0.017	(0.011	0.024)	0.010	(0.006	0.016)

Age sero-prevalence data were used to fit a catalytic conversion model [2] for data from RDT and filter paper for each combination of study area and antigen. As prevalence plots from Same indicated a recent drop in infection intensity, both previous and current estimates of sero-conversion rate are presented.

One advantage of serological assessment of malaria transmission intensity is the opportunity to investigate historical changes in the force of infection, which may be revealed by biphasic or multiphasic seroconversion rate (SCR) curves (a proxy for force of infection) [2]. In Korogwe district, the best fit for the model assumed a single SCR across the entire age range, whereas for Same district the best fit for both RDT-derived and FPBS-derived data required two separate SCRs to be fitted assuming a reduction in SCR approximately 10 years previously [11].

Together, these data suggest that RDTs and FPBSs are equally suitable sources of antibodies for estimating anti-malarial sero-conversion rates.

## Discussion

The aim of this study was to investigate the suitability of *P. falciparum *HRP-2 RDTs as matrices for collection of antibodies for malaria sero-epidemiology studies. Using previously validated immuno-assays and statistical analyses [1-3,11], we compared the recovery of anti-malarial antibodies from RDTs after storage under varying conditions for varying lengths of time and we compared data obtained from RDTs with data obtained from filter paper blood spots which had been collected and stored according to a previously validated protocol [3].

Encouragingly, anti-malarial antibodies within RDTs were well preserved over periods of up to 70 days and it is likely that acceptable data could be obtained from RDTs stored for longer periods. Optimal preservation of anti-malarial antibodies was observed for RDTs stored at 4°C, as previously observed for filter-paper blood spots [3]. This was expected because degradative processes and bacterial growth are likely to be temperature- and humidity-dependent. Data presented here indicate that samples were equally well preserved at moderate humidity (i.e. RDTs thoroughly air-dried but stored without desiccant) and at low humidity (i.e. when desiccant was used). This differs from observations for filter paper blood-spots which were observed to be best stored desiccated [3]. This difference between filter paper and RDTs could be explained by preservatives added to RDTs during their manufacture, and/or it is possible that immunoglobulins were well-preserved in desiccated RDTs but eluted poorly. We suggest that the most important consideration for operational storage and transportation of RDTs for malaria sero-epidemiology is to prevent humidity-dependent sample degradation. Thorough air-drying might not always be a realistic option for field studies, particularly during rainy or humid conditions. For this reason, it is likely that the best way to ensure that RDTs remain dry under realistic operational conditions is to store them with desiccant. Data from the field study presented here, where RDTs were stored in air tight containers with dessicant for up to two weeks before being transported to a cold room, suggest that appropriate collection and storage of RDTs can be readily achieved in a field setting.

Antibody titres from paired filter paper and RDT samples were clearly correlated, albeit with a moderate degree of scatter and a tendency for RDT-derived titres to be greater than those from filter paper blood-spots. These inaccuracies may reflect differences in the volume of blood loaded onto the RDTs. The blood content of filter paper blood-spots is highly consistent because blood spreads uniformly across the filter paper and is extracted from a fixed area (determined by the diameter of the hole punch used to cut out the spot) constituting a defined, highly reproducible blood volume dependent on the weight of the filter paper [3]. For RDTs this approach is not possible, and the precision of RDT-based serology is limited by the accuracy with which blood is initially applied to the test. In this investigation, RDTs were assumed to contain 5 μl of blood, because this is the amount recommended to be added to devices by their manufacturer. In practice, where RDTs are used by busy health-care workers, a degree of inaccuracy in the amount of blood added is perhaps unsurprising.

By more accurately dispensing blood onto RDTs, it is likely that the observed bias towards RDT-derived titres exceeding those from FPBSs could be removed. In practice, though, this is unlikely to be important for estimating sero-conversion rates because sero-positivity cut-offs can be generated using mixture modelling [3,13]. This assumes that antibody titres are distributed as a mixture of two (or possibly more) partially overlapping normal (Gaussian) distributions. One of these distributions represents antibody-negative individuals; its mean and standard deviation are then used to define the cut-off for negativity. In this investigation, cut-off values for RDT samples were higher than for filter paper samples and, as a consequence, there was strong agreement in serological status (i.e. sero-negative or sero-positive) for both MSP-1_1–19 _and AMA-1. There appeared to be no systematic distribution of discordant values with either age or between the low and high malaria transmission areas. Sero-conversion rates, derived from the age-specific sero-prevalence data, were highly correlated for RDT- and filter paper-derived samples for both antigens and in both study sites. Therefore, serological estimation of malaria transmission intensities from RDTs reproduced results obtained from filter paper blood-spots.

The primary function of RDTs remains to provide diagnostic information to facilitate decisions on drug administration in the context of clinical service provision. However, RDTs are increasingly being used by researchers for rapid assessment of *P. falciparum *parasite prevalence [14] and it is in this context that re-using RDTs to obtain serological data may become a useful additional application. Recovery of immunoglobulins from RDTs which have already been used for diagnosis or parasite detection will remove the need for additional blood samples to be taken, hence saving time, money and effort, in addition to minimizing distress and discomfort for subjects. Therefore, RDTs are an attractive source of immunoglobulins for estimating sero-prevalence and to assess prior exposure to malaria.

## Conclusion

This study shows that malarial RDTs provide stable matrices from which anti-malarial antibodies can be eluted and assayed for estimation of sero-prevalence and sero-conversion rates. The ease of collection, storage and elution of antibodies from RDTs, when combined with the information on parasite prevalence that RDTs provide, suggest these devices can be very useful for sample collection in serological studies of malaria endemicity.

## Competing interests

The authors declare that they have no competing interests.

## Authors' contributions

GW carried out the laboratory assays, performed initial analysis and wrote the first draft of the manuscript. CM & LS contributed to laboratory analysis and sample collection. GM and HR contributed to sample collection and data interpretation. JC produced antigens, advised on assay design and data interpretation. PC, ER & CD conceived and designed the study and contributed substantially to technical interpretation and writing of the manuscript. All authors have seen and commented on the manuscript.
